# Supplementary material for: Relationship between the Dynamics of Flavor Compounds and Microbial Succession in the Natural Fermentation of Zhalajiao, a Popular Traditional Chinese Fermented Chili Paste
Source: Foods. 2023 Oct 20;12(20):3849. doi: 10.3390/foods12203849 (PMC10606277; doi:10.3390/foods12203849)
Supplement: Supplementary file 1 [file foods-12-03849-s001.zip › foods-2593960-supplementary.pdf]

Table S1. Changes of volatile compounds (μg/kg) during the fermentation of Zhalajiao.

No.	CAS	Compounds (μg/kg)	RT（min）	LRI	D0	D7	D15	D22	D30	D45	D60	D90
		**Esters (mg/kg)**			5.22	7.68	8.10	8.34	1.03	41.72	42.89	5.94
1	105-54-5	Ethyl butyrate	4.717	769	nd	nd	nd	nd	137.87±8.98	876.56±277.12	938.81±43.43	118±16.72
2	628-63-7	n-Pentyl acetate	6.117	881	nd	nd	nd	nd	706.1±46.48	1588.79±167.61	1632.04±125.17	515.26±28.33
3	539-82-2	Ethyl n-valerate	6.533	904	nd	nd	nd	nd	24.14±1.41	329.18±27.03	501.03±79.31	131.28±1.68
4	25415-67-2	Pentanoic acid,4-methyl-, ethyl ester	7.692	967	nd	nd	nd	nd	74.05±3.83	373.16±45.90	685.2±57.69	23.77±1.79
5	123-66-0	Ethyl caproate	8.267	997	15±1.47	189.68±32.33	292.56±12.60	377.55±18.49	461.05±18.95	1429.51±415.60	333.51±34.41	284.53±16.99
6	142-92-7	Hexyl acetate	8.508	1010	nd	nd	85.59±6.07	105.95±9.94	539.33±57.98	1153.54±73.06	95.54±4.02	140.7±4.84
7	106-30-9	Ethyl heptanoate	9.917	1095	nd	nd	nd	nd	111.96±10.81	163.2±5.26	70.22±4.38	43.48±5.34
8	2349-07-7	Hexyl isobutyrate	10.15	1110	nd	115.31±11.79	76.36±3.06	95.84±5.26	173.02±8.53	392.96±8.08	30.53±1.02	175.59±23.3
9	68039-26-9	Butanoic acid,2-methyl-, pentyl ester	10.575	1137	nd	nd	nd	nd	7.26±1.62	32.63±6.09	24.55±2.63	24.05±1.98
10	93-89-0	Ethyl benzoate	11.15	1179	nd	nd	nd	nd	40.72±4.36	88.89±7.39	143.34±12.56	46.08±3.41
11	106-32-1	Octanoic acid, ethyl ester	11.467	1194	nd	nd	300.94±25.25	343.33±22.24	438.19±42.79	945.85±29.39	529.88±35.47	142.28±20.36
12	10032-13-0	Butanoic acid,3-methyl-, hexyl ester	11.625	1210	1673.52±100.16	1217.27±202.8	856.31±53.1	464.91±27.68	549.37±44.13	1636.44±161.37	710.71±16.47	312.63±22.59
13	2198-61-0	Hexanoic acid,3-methyl-,butyl ester	11.767	1219	nd	nd	nd	nd	30.01±1.69	135.95±3.57	138.4±20.36	28.97±0.72
14	1117-59-5	Pentanoic acid, hexylester	12.15	1275	nd	nd	nd	nd	19.55±2.54	42.66±2.06	49.92±6.55	43.02±1.65
15	118-61-6	Benzoic acid,2-hydroxy-, ethyl ester	12.567	1295	nd	129.05±1.06	146.8±7.7	208.62±19.87	245.96±40.32	395.68±25.12	358.16±27.19	49.15±4.32
16	7367-84-2	(*Z*)-4-decenoic acid, ethyl ester	13.592	1359	541.14±87.22	595.58±51.93	655.67±32.83	788.87±38.62	1031.16±88.91	3444.37±241.29	2371.5±104.17	598.75±29.7
17	6378-65-0	Hexanoic acid, hexylester	13.208	1370	nd	489.43±59.83	404.44±19.14	531.72±11.82	570.16±31.35	1579.6±93.18	863.6±41.49	406.81±39.14
18	110-38-3	Decanoic acid, ethyl ester	13.883	1392	nd	166.07±18.58	176.08±9.43	211.23±12.9	257.58±12.01	797.66±65.72	574.52±24.48	93.34±2.91
19	627-90-7	Undecanoic acid, ethyl ester	15.708	1455	nd	nd	nd	nd	164.32±17.03	520.17±35.84	493.45±35.54	72.1±4.22
20	106-33-2	Ethyl laurate	18.783	1592	153.92±21.98	146.61±18.77	199.63±5.85	193.01±0.78	300.26±17.08	1130.99±156.96	1461.41±251.21	171.76±9.87
21	28267-29-0	Ethyln-tridecanoate	21.2	1692	nd	nd	nd	nd	21.84±3.38	556.41±13.37	219.31±28.66	49.47±4.68
22	124-06-1	Tetradecanoic acid,ethyl ester	22.767	1792	1597.88±343.44	1283.73±267.51	1469.52±152.9	1474.5±136.36	1458.06±214.66	6643.37±663.79	9041.81±572.61	596.16±52.77
23	41114-00-5	Pentadecanoic acid,ethyl ester	25.225	1883	nd	nd	nd	nd	153.43±18.33	670.02±35.88	128.61±28.18	25.89±3.82

24	54546-22-4	9-Hexadecenoic acid,ethyl ester	27.983	1987	nd	nd	nd	nd	34.61±4.43	81.4±5.19	432.56±21.81	93.62±2.87
25	628-97-7	Hexadecanoic acid,ethyl ester	27.642	1992	45.14±6.93	2389.98±137.26	2500.06±216.3	2532.33±260.26	2016.65±330.13	11234.15±884.91	15199.59±1647.49	1011.83±64.39
26	112-63-0	Octadecanoic acid,methyl ester	30.95	2098	nd	nd	nd	nd	nd	334.43±9.37	323.68±30.81	84.97±9.76
27	111-62-6	9-Octadecenoic acid(9Z)-, ethyl ester	32.442	2173	643.47±149.38	331.41±23.96	317.16±8.75	332.19±13.31	201.62±13.83	616.15±52.95	301.55±7.12	230.88±24.61
28	544-35-4	Linoleic acid, ethyl ester	32.283	2190	551.45±6.76	623.87±44.47	617.36±36.2	675.17±44.68	264.89±17.16	4525.6±431.56	5239.93±300.79	425.48±24.43
		**Alcohols (mg/kg)**			0	0	0	0	1.03	1.18	0.88	0.10
29	107-88-0	1,3-Butanediol	5.017	819	nd	nd	nd	nd	1030.2±129.91	1175.01±76.2	880±13.19	103.65±6.46
		**Aldehydes (mg/kg)**			0	0	0	0	0	0.15	0.24	0.06
30	18829-56-6	(2E)-2-Nonenal	10.958	1166	nd	nd	nd	nd	nd	148.86±2.83	243.68±23.33	56.02±1.44
		**Acids (mg/kg)**			0	0	0	0	0.26	1.27	1.10	0.41
31	111-14-8	Heptanoic acid	9.5	1073	nd	nd	nd	nd	36.56±1.87	133.02±17.7	198.99±14.49	66.06±4.64
32	124-07-2	Octanoic acid	11.05	1169	nd	nd	nd	nd	110.18±4.14	274.88±15.43	163.16±22.55	104.74±16.37
33	1560-97-0	Nonanoic acid	12.442	1265	nd	nd	nd	nd	17.81±1.11	670.87±29.33	442.19±40.48	124.12±11.47
34	31295-56-4	Decanoic acid	13.45	1332	nd	nd	nd	nd	53.23±2.66	76.27±1.83	47.69±3.05	23.63±1.26
35	143-07-7	Lauric acid	17.916	1557	nd	nd	nd	nd	45.01±2.96	111.26±6.04	243.92±40.64	86.72±5.16
		**Terpenes (mg/kg)**			24.88	2.19	1.95	3.49	4.94	16.01	14.88	6.42
36	123-35-3	Myrcene	8.15	993	nd	nd	nd	nd	178.25±18.37	393.97±20.99	279.14±50.08	387.37±14.74
37	5989-27-5	(+)-Limonene	8.8	1033	14133.1±3082.24	55.23±3.91	59.06±1.3	215.38±20.82	123±4.01	820.53±75.33	1678.31±224.03	673.06±28.87
38	13877-91-3	Ocimene	8.917	1059	2160.5±374.99	341±23.14	357.68±10.32	520.83±36.53	501.15±39.7	1593.74±190.41	958.72±36.66	308.06±10.27
39	78-70-6	Linalool	9.942	1100	272.04±43.2	343.29±31.31	355.01±15.95	555.28±35.16	708.29±37.78	2529.34±93.1	2476.01±255.39	1652±91.18
40	562-74-3	4-Terpineol	11.275	1187	nd	nd	nd	nd	37.61±1.91	167.97±10.75	351.32±6.65	250.38±4.75
41	515-13-9	β-Elemene	14.508	1396	1582.97±222.85	288.79±26.41	246.7±15.34	327.43±10.43	368.61±20.01	1120.18±80.55	905.79±60.72	154.51±4.26
42	25246-27-9	allo-Aromadendrene	14.825	1411	nd	nd	nd	nd	11.13±2.26	27.04±0.34	91.54±2.99	28.77±0.49
43	475-20-7	(+)-Longifolene	15.017	1421	nd	nd	nd	nd	7.85±0.96	66.44±3.34	40.88±3.61	13.46±3.05
44	469-61-4	α-Cedrene	15.133	1427	nd	nd	96.56±1.83	131.66±0.83	258.1±6.61	731.44±21.28	750.66±49.13	345.58±20.94
45	28973-97-9	(E)-β-farnesene	15.642	1451	nd	nd	nd	nd	61±3.14	182.43±5.28	217.31±3.45	66.44±4.33

46	17283-81-7	β-Ionone	16.308	1482	nd	nd	nd	nd	272.54±17.64	965.86±62.4	935.21±33.46	204.22±15.94
47	3853-83-6	α-Himachalene	16.483	1488	3029.52±321.5	293.79±10.93	406.18±30.9	752.91±70.44	857.28±19.93	1624.71±54.06	260.16±21.85	104.81±5.13
48	87-44-5	β-Caryophyllene	16.408	1492	3165.02±252.72	773.76±58.3	304.62±18.31	843.23±29.64	526.45±25.85	3098.14±120.74	2428.62±133.43	1499.78±200.07
49	3691-12-1	α-Guaiene	16.758	1507	nd	nd	nd	nd	93.92±4.82	218.94±3.31	260.16±22.39	101.98±2.28
50	1461-03-6	β-Himachalene	16.842	1511	nd	nd	nd	nd	180.23±13.8	464.64±20.68	785.18±61.32	42.15±3.37
51	16982-00-6	α-Curcumene	17.033	1519	535.29±66.05	97.11±2.82	120.97±5.52	139.32±20.14	225.56±7.44	739.32±40.52	762.43±14.21	158.72±6.64
52	20129-39-9	(+)-δ-Cadinene	17.258	1526	nd	nd	nd	nd	30.6±0.66	73.04±5.48	163.1±11.19	71.84±4.1
53	77-53-2	(+)-Cedrol	19.383	1617	nd	nd	nd	nd	497.7±12.88	1195.75±63.79	1531.69±123	352.99±15.19
		**Pyrazines (mg/kg)**			0	0	0	0	0.03	0.11	0.18	0.02
54	24683-00-9	2-Isobutyl-3-methoxypyrazine	11.142	1199	nd	nd	nd	nd	29.9±0.83	108.08±10.29	178.27±10.93	22.48±2.05
		**Alkanes (mg/kg)**			6.01	3.43	3.41	4.37	5.00	15.18	21.87	3.72
55	1560-96-9	Tridecane, 2-methyl-	13.942	1361	1573.89±239.94	864.31±63.07	880.83±31.54	1168.48±331.86	1256.89±199.71	4854.67±191.32	2463.27±129.09	742.04±50.59
56	629-59-4	tetradecane	14.583	1402	497.15±53.05	253.6±11.68	249.9±14.73	301.18±15.68	344.89±32.92	906.7±32.7	657.88±28.05	142.86±13.1
57	1560-95-8	Tetradecane, 2-methyl-	15.875	1462	1565.12±271.19	915.43±82.82	955.29±58.96	1193.559±206.76	1395.26±224.29	2710.25±289.35	8504.14±485.29	910.54±28.09
58	629-62-9	pentadecane	16.658	1505	152.83±24.78	262.68±21.76	268.79±12.46	366.25±40.89	446.53±12.36	1438.61±154.77	1656.43±110.66	304.01±3.4
59	1560-93-6	Pentadecane, 2-methyl-	18.108	1544	283.89±20.95	339.94±15.41	354.73±30.56	411.53±17.75	522.91±34.96	1895.28±32.52	2054.65±184.44	331.2±8.9
60	544-76-3	Hexadecane	18.967	1603	123.37±10.39	385.81±22.02	365.15±12.41	466.7±29.53	431.79±46.6	1347.43±79.53	2117.02±188.3	399.27±36.97
61	1921-70-6	Pentadecane,2,6,10,14-tetramethyl-	20.092	1646	nd	nd	nd	nd	80.01±4.01	223.68±27.74	1076.86±61.89	266.93±6.56
62	544-76-3	Hexadecane, 2-methyl-	20.5	1663	594.81±29.94	84.74±2.31	86.16±5.36	104.85±4.57	133.68±9.68	405.71±7.76	789.61±27.86	147.46±11.32
63	629-78-7	heptadecane	21.408	1700	914.92±75.05	220.92±25.52	174.27±12.78	258.07±16.61	217.95±12.79	895.31±10.72	1501.69±185.65	287.35±35.38
64	6418-44-6	Heptadecane, 3-methyl-	23.15	1771	nd	nd	nd	nd	20.08±2.08	47.93±1.71	152.15±6.76	58.22±5.02
65	638-36-8	Hexadecane,2,6,10,14-tetramethyl-	24.042	1742	nd	nd	nd	nd	95.67±3.38	218.65±28.71	448.6±6.56	43.32±2.54
66	593-45-3	Octadecane	24.008	1806	301.67±11.67	98.84±7.2	79.07±4.14	99.21±5.83	56.56±2.26	235.03±23.9	448.6±16.15	90.45±6.77
		**Total (mg/kg)**			36.11	13.30	13.46	16.19	21.3	75.61	82.04	16.67
